# Total kidney and liver volume is a major risk factor for malnutrition in ambulatory patients with autosomal dominant polycystic kidney disease

**DOI:** 10.1186/s12882-016-0434-0

**Published:** 2017-01-14

**Authors:** Hyunjin Ryu, Hyosang Kim, Hayne Cho Park, Hyunsuk Kim, Eun Jin Cho, Kyu-Beck Lee, Wookyung Chung, Kook-Hwan Oh, Jeong Yeon Cho, Young-Hwan Hwang, Curie Ahn

**Affiliations:** 1 Department of Internal Medicine, Seoul National University College of Medicine, Seoul, 110-744 Republic of Korea; 2 Department of Internal Medicine, Asan Medical Center, Seoul, Korea; 3 Department of Internal Medicine, Armed Forces Capital Hospital, Seongnam, Korea; 4 Department of Internal Medicine, Kangbuk Samsung Hospital, College of Medicine, Sungkyunkwan University, Seoul, Korea; 5 Department of Internal Medicine, Gil Medical Center, Gacheon University, Incheon, Korea; 6 Department of Radiology, Seoul National University Hospital, Seoul, Korea; 7 Department of Internal Medicine, Eulji General Hospital, Seoul, Korea

**Keywords:** Autosomal-dominant polycystic kidney disease, Chronic kidney disease, Gender, Malnutrition, Polycystic liver disease

## Abstract

**Background:**

In patients with autosomal dominant polycystic kidney disease (ADPKD), malnutrition may develop as renal function declines and the abdominal organs become enlarged. We investigated the relationship of intra-abdominal mass with nutritional status.

**Methods:**

This cross-sectional study was performed at a tertiary hospital outpatient clinic. Anthropometric and laboratory data including serum creatinine, albumin, and cholesterol were collected, and kidney and liver volumes were measured. Total kidney and liver volume was defined as the sum of the kidney and liver volumes and adjusted by height (htTKLV). Nutritional status was evaluated by using modified subjective global assessment (SGA).

**Results:**

In a total of 288 patients (47.9% female), the mean age was 48.3 ± 12.2 years and the mean estimated glomerular filtration rate (eGFR) was 65.3 ± 25.3 mL/min/1.73 m^2^. Of these patients, 21 (7.3%) were mildly to moderately malnourished (SGA score of 4 and 5) and 63 (21.7%) were at risk of malnutrition (SGA score of 6). Overall, patients with or at risk of malnutrition were older, had a lower body mass index, lower hemoglobin levels, and poorer renal function compared to the well-nourished group. However, statistically significant differences in these parameters were not observed in female patients, except for eGFR. In contrast, a higher htTKLV correlated with a lower SGA score, even in subjects with an eGFR ≥45 mL/min/1.73 m^2^. Subjects with an htTKLV ≥2340 mL/m showed an 8.7-fold higher risk of malnutrition, after adjusting for age, hemoglobin, and eGFR.

**Conclusions:**

Nutritional risk was detected in 30% of ambulatory ADPKD patients with relatively good renal function. Intra-abdominal organomegaly was related to nutritional status independently from renal function deterioration.

**Electronic supplementary material:**

The online version of this article (doi:10.1186/s12882-016-0434-0) contains supplementary material, which is available to authorized users.

## Background

Malnutrition increases mortality, morbidity, and the duration of the hospital stay in various clinical settings, including inpatient settings in general as well as in liver failure and cancer patients [1]. In chronic kidney disease (CKD), the prevalence of malnutrition increases to 30–40% of patients, and protein-energy malnutrition is one of the strongest predictors of morbidity and mortality [2, 3]. In previous studies, nutritional markers such as serum albumin, creatinine, body mass index (BMI), and subjective global assessment (SGA) score were independent predictors of death and treatment failure in CKD [4, 5]. Pre-transplant nutritional status also influences the outcomes of kidney transplantations [6]. Therefore, efforts have been made to establish guidelines for properly assessing the nutritional status of CKD patients and intervening to improve their outcomes [7]. However, the value of nutritional markers in the early stage of CKD was not meticulously evaluated in patients with early stages of CKD.

Autosomal dominant polycystic kidney disease (ADPKD) is the most common hereditary kidney disease, and can progress to end-stage renal disease (ESRD) as kidney cysts grow. The prevalence of liver cysts in ADPKD patients was 58% in patients aged 15–24 years and up to 94% of patients older than 35 years [8]. Many uncontainable complications can develop as cysts grow to cause massive organomegaly. From previous study, mass effect due to organomegaly was reported to cause pressure related symptoms (46.5%), pain (58.8%), gastrointestinal symptoms (32.4%) and obstructive complications which can lead to leg edema (20.4%), ascites (16.6%) and infection (3.1%) [9]. In these patients, pressure effects from the enlarged organs may also result in poor oral intake and eventually malnutrition. Occasionally, massive organomegaly requires volume reduction interventions to relieve symptoms and to improve the patient’s quality of life [10].

In ADPKD, the mass effects from increased kidney and liver volume may aggravate malnutrition, even in the early stages of kidney disease [11]. Therefore, assessment of nutritional status even in the early stages of ADPKD with significant organomegaly is advised to ensure timely interventions that result in the subsequent improvement of clinical outcomes as in polycystic liver disease patients [11, 12]. However, traditional anthropometric parameters, such as body weight and BMI, are of limited value because of the fluid-filled kidneys and liver. In this study, we evaluated the nutritional status of ambulatory ADPKD patients using SGA as a standard method, and identified intra-abdominal organ volume as an independent risk factor for malnutrition.

## Methods

### Patient population

ADPKD patients who visited polycystic kidney disease clinic in Seoul National University Hospital from December 2013 to March 2014 were included in this study. Patients of age 18 years and older who agreed to participate in the study were included. Abdominal computed tomography (CT) scan on ADPKD patients were taken every other year for clinical purpose as a standardized evaluation protocol during the outpatient clinic [13]. Patients with active cancer, active infection, CKD stage 5 at the time of enrollment, ESRD treated with renal replacement therapy, or a history of volume-reductive therapies of liver (transarterial embolization, liver resection or transplantation) due to severe polycystic liver disease were excluded. Electronic medical records were reviewed retrospectively and 31 patients were identified who met exclusion criteria.

Since this study was a cross-sectional one using clinical data, and it did not involve further invasive intervention, treatment, or costs to patients, the study received a consent exemption and it was approved by the Institutional Review Board of Seoul National University Hospital (H-1407-083-594). The patient’s record was de-identified and analyzed anonymously. This study was performed in accordance with the Declaration of Helsinki.

### Subjective global assessment and clinical data collection

The SGA score is a method of nutritional assessment that has been well validated in various settings and is based on a clinical history and physical examination. Nutritional assessment has been validated in CKD patients as a predictor of complications and outcomes [10–13]. Based on these results, SGA has been recommended in the Kidney Disease Outcomes Quality Initiative guidelines as a nutritional assessment tool, especially for CKD patients [7]. SGA is frequently used as a reference method for evaluating new nutritional assessment techniques.

The modified SGA, which has been validated in many studies of CKD patients [14–17], was performed to evaluate the nutritional status of ADPKD patients according to the standardized protocol in our clinic from December 2013. A well-trained internist performed SGA to ensure consistency. SGA consists of a medical history (weight changes, dietary intake, gastrointestinal symptoms, functional capacity, and comorbidities related to nutritional needs) and a physical examination. In detail, a clinician inspected subcutaneous fat below the eye, triceps or biceps area or at chest area, and examined the temples, clavicles and the back of the hands for muscle wasting. The presence of edema or ascites was assessed by physical examination. Based on these components, a clinician uses a seven-point scale to reflect an overall judgment of the patient’s nutritional status. The SGA score was interpreted as follows: 7, well nourished; 6, at risk; 5, mildly malnourished; 3–4, moderately malnourished; and 1–2, extremely malnourished. Laboratory tests, including serum hemoglobin, creatinine, total protein, albumin, and total cholesterol were simultaneously performed. Estimated glomerular filtration rates (eGFR) were calculated by the Chronic Kidney Disease Epidemiology equation, using isotope dilution mass spectrometry-traceable creatinine [18].

### Volume measurement of kidneys and liver

In our polycystic kidney disease clinic, abdominal CT scans were taken every other year. A latest abdominal CT scan at the time of nutritional assessment was used to measure total liver volume (TLV) and total kidney volume (TKV). The mean time interval between the CT scan and the nutritional assessment was 12.5 ± 12.6 months. TLV was calculated by adding the product of slice thickness and the area measured on a set of contiguous images generated by CT using Rapidia 2.8 CT software (INFINITT Healthcare Co. Ltd, Seoul, Korea). TKV was estimated by using the ellipsoid method [19]. Height-adjusted TLV (htTLV, mL/m) and height-adjusted TKV (htTKV, mL/m) were used in this study. Height-adjusted total kidney and liver volume (htTKLV, mL/m) was defined as the sum of the htTLV and htTKV values.

### Statistical analyses

For statistical analysis between genders, we used student *t*-test for variables with a normal distribution and used Mann-Whitney test for variables with a non-normal distribution (height, weight, protein, albumin, htTLV, htTKV, and htTKLV). For SGA scores, all patients were classified into three groups: mildly to moderately malnourished (an SGA score of 4–5), at risk (an SGA score of 6), and well nourished (an SGA score of 7), because none had SGA score less than 4 [20]. For statistical analysis, we used linear association test or Jonckheere-Terpstra test to analyze the *p*-for trend among three SGA groups. *P-*values <0.05 were considered to indicate statistical significance.

Receiver operating characteristic (ROC) curve analysis was used to evaluate htTKLV as a discriminating parameter for malnutrition (SGA score ≤5), in contrast with the well-nourished group (score 7). In this analysis we excluded patients with SGA score of 6 to analysis the effect of htTKLV on definite malnutrition. The Youden index was used to determine the optimal cutoff value. Binominal logistic regression was used to test the significance of the htTKLV threshold after adjusting for age, hemoglobin, and eGFR. All statistical analyses were conducted using SPSS version 22 (IBM Corporation, Armonk, NY, USA) and MedCalc for Windows version 14 (MedCalc Software, Ostend, Belgium).

## Results

### Baseline characteristics

A total of 288 patients were included in the analysis, of whom 138 (47.9%) were female. The mean age was 48.3 ± 12.2 years, with no significant difference according to gender. The mean SGA scores were similar (6.7 ± 0.6 vs. 6.6 ± 0.6, *p* = 0.197) between genders. The mean eGFR were 65.3 ± 25.3 mL/min/1.73 m^2^ and eGFR was higher in female patients (62.3 ± 24.5 mL/min/1.73 m^2^ vs. 68.5 ± 25.8 mL/min/1.73 m^2^, *p* = 0.035). There was no significant difference in albumin level between genders. The distribution of CKD stages was as follows: 52 patients (18.1%) were in stage 1 CKD, 116 (40.3%) were in stage 2, 53 (18.4%) were in stage 3A, 46 (16.0%) were in stage 3B, and 21 (7.3%) were in stage 4. The distribution of CKD showed no difference between genders. Two hundred thirty-eight patients (82.6%) had hypertension and 227 patients (78.8%) had liver cysts. Male had higher prevalence of hypertension (male; 134 patients, 89.3% and female; 104 patients, 75.4%, *p* = 0.002) and lower prevalence of liver cysts. (male; 101 patients, 67.3% and female 126 patients, 91.3%, *p* < 0.001). Also in order to evaluate the mass effect of kidney and liver volume on nutritional status, TKV and TLV were measured using CT scans and adjusted for height. Overall, the median htTKLV was 1776 mL/m with interquartile range (IQR) 1361–2381 mL/m, the median htTKV was 697 mL/m (IQR 415–1133 mL/m), and the median htTLV was 977 mL/m (IQR 819–1196 mL/m). There were no significant difference between genders in htTKLV, htTKV and htTLV. With height unadjusted volume, TKLV and TLV did not showed statistical difference between genders (*p* = 0.076 and p.0.255 respectively). Only TKV showed difference between gender (*p* = 0.003), however the difference disappeared after adjusting with height (*p* = 0.057) (Additional file 1: Table S1).

### Nutritional status of subjects

Mild to moderate malnutrition was detected in 7.3% of all patients. Only two patients (0.7%) had an SGA score of 4, and 19 patients (6.6%) had a score of 5. Sixty-three patients (21.9%) were at risk of malnutrition (a score of 6), and 204 (70.8%) were well nourished (a score of 7). No statistical difference was observed in the distribution of SGA scores between genders (Table 1).

**Table 1:** Baseline patient characteristics according to nutritional status as evaluated by SGA

Parameters	Mildly to moderately malnourished (SGA 4 and 5)	At risk (SGA 6)	Well nourished (SGA 7)	*P* for trend
Number of patients	21 (7.3%)	63 (21.9%)	204 (70.8%)	
Female	9 (42.9%)	37 (58.7%)	92 (45.1%)	0.148
Age (years)	53.4 ± 11.1	52.7 ± 12.6	46.4 ± 11.7	<*0.001*
Height (cm)	164.0 ± 7.9	163.4 ± 7.9	167.6 ± 9.8	*0.002*
Weight (kg)	59.1 ± 8.7	62.3 ± 9.7	67.0 ± 12.3	*0.001*
BMI (kg/m^2^)	22.0 ± 2.7	23.3 ± 2.6	23.7 ± 2.9	*0.044*
Hemoglobin (g/dL)	12.8 ± 1.1	13.2 ± 1.4	13.7 ± 1.5	*<0.001*
eGFR (mL/min/1.73 m^2^)	51.3 ± 23.2	57.1 ± 24.9	69.2 ± 24.6	<*0.001*
Protein (g/dL)	7.4 ± 0.4	7.2 ± 0.4	7.3 ± 0.4	0.328
Albumin (g/dL)	4.4 ± 0.3	4.3 ± 0.3	4.4 ± 0.4	0.377
Total cholesterol (mg/dL)	180.3 ± 30.2	175.8 ± 27.7	177.4 ± 26.0	0.654
Presence of hypertension	21 (100%)	55 (87.3%)	162 (79.4%)	*0.011*
Presence of liver cysts	17 (81%)	55 (87.3%)	155 (76.0%)	0.160
TKLV (mL)	4,581 [2,698–7,899]	3,445 [2,642–4,415]	2,808 [2,241–3,695]	*<0.001*
TKV (mL)	2,089 [817–2,801]	1,443 [913–2,070]	1,059 [638–1,628]	*<0.001*
TLV (mL)	2,039 [1,336–4,728]	1,659 [1,352–2,200]	1,636 [1,337–1,951]	0.120
htTKLV (mL/m)	2,622 [1,719–4,906]	2,147 [1,535–2,687]	1,649 [1,311–2,151]	*<0.001*
htTKV (mL/m)	1,282 [524–1,574]	903 [559–1,246]	640 [394–955]	*<0.001*
htTLV (mL/m)	1,298 [830–2,831]	1,023 [816–1,322]	957 [819–1,147]	*0.036*

TKLV, TKV, TLV, htTKLV, htTKV and htTLV are shown in median and interquartile range

*BMI* body mass index, *CKD* chronic kidney disease, *eGFR* estimated glomerular filtration rates, *htTKV* height-adjusted total kidney volume, *htTKLV* height-adjusted total kidney and liver volume, *htTLV* height-adjusted total liver volume, *SGA* subjective global assessment, *TKV* total kidney volume, *TKLV* total kidney and liver volume, *TLV* total liver volume

**Fig. 1:**
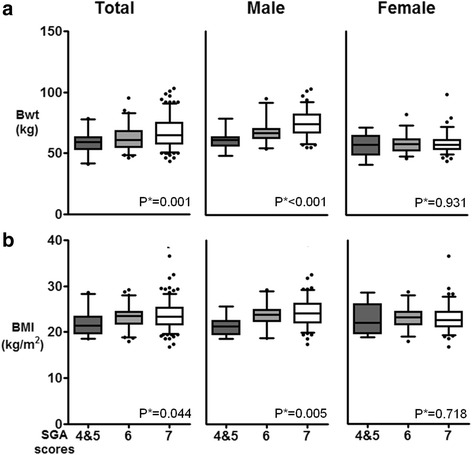
Correlations between the SGA score and the anthropometric nutritional parameters. **a** body weight (Bwt) and **b** body mass index (BMI). P*; *p* for trend, SGA; subjective global assessment, SGA 7, well-nourished; SGA 6, at risk; SGA 5, mildly malnourished; SGA 3–4, moderately malnourished

Patients with malnutrition (SGA 4–5) or at risk for malnutrition (SGA 6) were older than the well-nourished group (SGA 7) (mean age, 53.4 ± 11.1 years vs. 52.7 ± 12.6 years vs. 46.4 ± 11.7 years, respectively, p for trend <0.001). In terms of anthropometric parameters, weight (59.1 ± 8.7 kg vs. 62.3 ± 9.7 kg vs. 67.0 ± 12.3 kg, respectively, p for trend 0.001) and BMI (22.0 ± 2.7 kg/m^2^ vs. 23.3 ± 2.6 kg/m^2^ vs. 23.7 ± 2.9 kg/m^2^, respectively, p for trend 0.044) tended to show lower values in patients with lower SGA scores. However, this trend was observed only in male patients, and none of the anthropometric parameters showed statistically significant differences in female patients (Fig. 1).

**Fig. 2:**
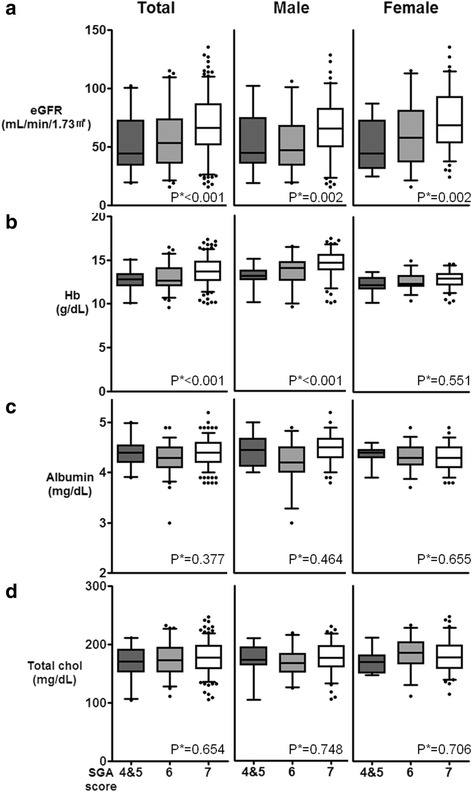
Correlations between the SGA score and the following laboratory markers. **a** estimated glomerular filtration rate (eGFR), **b** hemoglobin (Hb), **c** albumin, and **d** total cholesterol (total chol). P*; *p* for trend, SGA; subjective global assessment, SGA 7, well-nourished; SGA 6, at risk; SGA 5, mildly malnourished; SGA 3–4, moderately malnourished

With regard to laboratory parameters, decreased eGFR values (51.3 ± 23.2 mL/min/1.73 m^2^ vs. 57.1 ± 24.9 mL/min/1.73 m^2^ vs. 69.2 ± 24.6 mL/min/1.73 m^2^, respectively, *p* for trend <0.001) were related to lower SGA scores. Hemoglobin (12.8 ± 1.1 g/dL vs. 13.2 ± 1.4 g/dL vs. 13.7 ± 1.5 g/dL, respectively, *p* for trend <0.001) also showed differences according to SGA scores. This trend was seen only in male patients, but not in female patients. Serum total cholesterol, total protein, and albumin levels showed no statistically significant differences by SGA score (Table 1, Fig. 2).

Hypertension showed higher prevalence in lower SGA groups (100, 87.3 and 79.4% respectively, *p* for trend = 0.011). However there was no statistical difference in liver cyst prevalence among SGA groups (*p* for trend = 0.16) (Table 1).

When we compared various data between SGA score of 4 and 5 (malnutrition) with 6 and 7 (at risk or well nourished), similar results were obtained (Additional file 1: Table S2).

### htTKLV was associated with SGA independently from eGFR

**Fig. 3:**
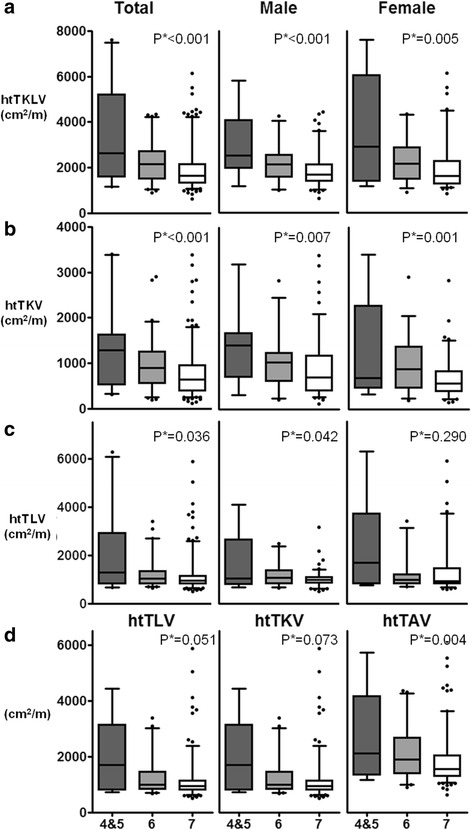
Correlations between the SGA score and abdominal volume. **a** height-adjusted total kidney and liver volume (htTKLV), **b** height-adjusted total kidney volume (htTKV), and **c** height-adjusted total liver volume (htTLV); **d** correlation between SGA score and abdominal volume in subjects with an eGFR ≥45 mL/min/1.73 m^2^. P*; *p* for trend, SGA; subjective global assessment, SGA 7, well-nourished; SGA 6, at risk; SGA 5, mildly malnourished; SGA 3–4, moderately malnourished

Lower SGA scores corresponded to higher values of htTKLV (median 2622 mL/m; IQR 1719–4906 mL/m vs. median 2147 mL/m; IQR 1535–2687 mL/m vs. median 1649 mL/m; IQR 1311–2151 mL/m, respectively, *p* for trend <0.001), htTKV (median 1282 mL/m; IQR 524–1574 mL/m vs. median 903 mL/m; IQR 559–1246 mL/m vs. median 640 mL/m; IQR 394–955 mL/m, respectively, p for trend <0.001), and htTLV (median 1298 mL/m; IQR 830–2831 mL/m vs. median 1023 mL/m; IQR 816–1322 mL/m vs. median 957 mL/m; IQR 819–1147 mL/m, respectively, *p* for trend =0.036) (Ta﻿ble 1, Fig. 3).

**Fig. 4:**
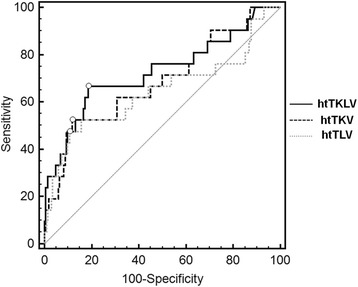
ROC curve of htTKLV, comparing SGA scores of 4 and 5 to 7. ROC; receiver-operating characteristics, SGA; subjective global assessment, SGA 7, well-nourished; SGA 6, at risk; SGA 5, mildly malnourished; SGA 3–4, moderately malnourished

ROC curve analysis was used to compare the volume parameters to identify a threshold predictive of malnutrition (an SGA of 4–5) over a state of being well nourished (an SGA score of 7). Since SGA score category 6 can be ambiguous due to the limitations of SGA itself, we constructed the ROC curve using the data of SGA score 7 (normal) and 4–5 (malnutrition). The area under the curve (AUC) of htTKLV was larger (0.727) than that of htTKV (0.687) and htTLV (0.645). The cut-off value for htTKLV was 2340 mL/m, with a sensitivity of 66.7% and a specificity of 81.4% (Fig. 4). By comparison, in an ROC curve analysis between an at-risk or malnourished state over a well-nourished state (an SGA of 4–6 vs. 7), similar but less significant results were obtained (AUC of htTKLV, htTKV, and htTLV were 0.658, 0.646, and 0.571, respectively), and the cut-off value for htTKLV was 2190 mL/m with a sensitivity of 53.6% and a specificity of 76.5% (data not shown).

**Fig. 5:**
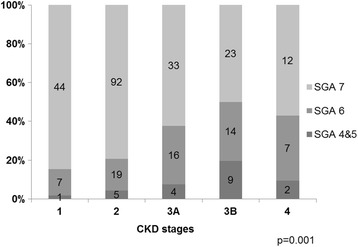
SGA score distribution according to CKD stages. *P*-values were obtained using the Fisher’s exact test. *Bars* indicate the percentage of patients in each category. CKD; chronic kidney disease, SGA; subjective global assessment

It is well known that the enlargement of the kidneys is closely related to renal insufficiency in ADPKD patients [21]. As expected, the eGFR fell as the SGA score decreased (Fig. 2), and the proportion of patients with lower SGA scores increased in our patients as the CKD stages increased from 1 to 3 (Fig. 5). When we stratified by CKD stage, even in stage 1 and 2 CKD, 15.4 and 20.9% of patients were either malnourished or at risk of malnutrition, respectively. Among stage 3 and 4 CKD patients, 43.4 and 42.8% were either malnourished or at risk of malnutrition, respectively. In patients with stage 4 CKD, the proportion of patients with a lower SGA score was slightly lower than among stage 3B CKD patients, which may have been due to the relatively small number of patients in stage 4 CKD or because we excluded patients with severe organomegaly who had already undergone surgical intervention.

In order to minimize the confounding effect of renal failure, subgroup analysis was performed in patients with an eGFR ≥45 mL/min/1.73 m^2^ (CKD stages 1–3A). In these patients, only htTKLV showed a significant association with SGA scores (Fig. 3d).

Using 2340 mL/m as the cut-off value of htTKLV based on ROC curve analysis, logistic regression analysis was used to estimate the odds ratio between the malnourished (an SGA score of 4–5) and the well-nourished group (an SGA score of 7) using variables that showed statistical significance among SGA groups. Patients with htTKLV ≥2340 mL/m showed a higher risk of malnutrition (an SGA score of 4–5) (odds ratio = 8.74, 95% confidence interval 3.30–23.13, *p* < 0.001), even after adjusting for age, hemoglobin, and eGFR.

## Discussion

Although previous studies have assessed the association of htTKV with renal function outcomes [22] and poor quality of life [23], this is the first study to assess the nutritional status of ADPKD patients and its relationship with htTKLV. In our previous study, an htTLV value >1600 mL/m was associated with an increase in pressure symptoms [9]. Therefore, we hypothesized that an enlarged liver and/or kidneys may exert a mass effect on the nearby areas of the gastrointestinal tract, causing gastrointestinal symptoms and eventually affecting the nutritional status of ADPKD patients. With this in mind, we measured htTKV and htTKLV, defining htTKLV to reflect the total mass effect of the enlarged kidneys and liver. We showed that htTKLV was the sole significant predictor of malnutrition after adjusting for other risk factors, including renal function. Even in subjects with relatively good renal function (eGFR ≥45 mL/min/1.73 m^2^), htTKLV was significantly associated with SGA scores of 4 and 5. Based on the ROC curve analysis and binominal logistic regression, htTKLV values ≥2340 mL/m, which is three times larger than the mean liver volume of healthy individuals [24], raised the risk of malnutrition by more than eightfold in ADPKD patients. When compared with htTLV and htTKV, htTKLV showed a closer relationship to malnutrition on the ROC curve, suggesting that total organ volume, instead of the size of each organ, may be responsible for the mass effect and the corresponding symptoms. However, since we excluded patients with severe polycystic liver disease who underwent surgical therapy (*n* = 16; mean htTLV, 5136 ± 2563 mL/m), the statistical association of htTLV with SGA scores could have been underestimated.

This study also shows that the prevalence of malnutrition in ADPKD should not be ignored. Most of patients had no malnutiriton (70.8%). However, even in outpatient clinic, 7.3% of patients were mildly to moderately malnourished (SGA scores of 4 and 5), and 21.9% of patients were at risk of malnutrition (an SGA score of 6). From previous studies, the prevalence of malnutrition in stage 4 and 5 CKD has been reported to be 20–30% [16, 25], and 10–60% of dialyzed patients have been found to have malnutrition (SGA score ≤ B by using conventional SGA or ≤5 by using modified SGA) [26]. Cuppari et al. [20] found that approximately 11% of patients with stage 2–5 CKD had protein-energy wasting (SGA ≤5) and 32% showed signs of protein-energy wasting (SGA 6). It is not proper to compare our data with those of Cuppari et al. [20], since most participants in their study were in the advanced stages of CKD (48.9% in stage 3 and 40.3% in stage 4), unlike our patient sample (58% in stage 1–2 CKD) (Fig. 5). Moreover, it is surprising that the prevalence of malnutrition risk (SGA ≤6) is up to 30% in patients treated in an ambulatory setting with relatively good renal function. We further analyzed whether these findings in ADPKD could be due to the increased volume of the kidneys and liver, which can cause various gastrointestinal symptoms and malnutrition even in the early stages of CKD. In our study, SGA score correlated with htTKLV even in the patients with relatively well preserved kidney function (eGFR ≥45 mL/min/1.73 m^2^ or CKD stages 1–3A).

Renal insufficiency itself can contribute to malnutrition and protein-energy wasting [27]. We also observed increased proportions of patients with malnutrition as the CKD stage advanced. The proportion of patients with a lower SGA score was slightly lower in stage 4 CKD than stage 3B patients, which may have been due to the relatively small number of patients in stage 4 CKD or because we excluded patients with severe organomegaly who had already undergone surgical intervention. In addition, when we analyzed parameters with SGA scores, most anthropometric or laboratory parameters that are widely used as markers for nutritional status failed to show an association with SGA scores, except for renal function. This finding that renal function was significantly related to SGA scores suggests regular assessment of nutritional status in ADPKD patients is needed as the disease progresses.

The association of parameters with SGA scores was different between genders. In male patients, old age, lower body weight, lower BMI, and lower hemoglobin levels were related to lower SGA scores, but these relationships were not seen in female patients. One of explanation would be the relatively less muscle mass in women compared to men, changes in body weight and BMI caused by malnutrition might be too small to be detected in our Asian women population. Moreover, enlarged cysts, ascites, or edema, which are frequent complications in ADPKD patients, may mask the reduction in muscle mass or fat proportion in the body. Laboratory parameters such as hemoglobin, total protein, albumin, or total cholesterol were not sensitive enough to detect changes in nutritional status during the early stages of malnutrition. Thus, further studies will be needed to confirm this finding in other cohorts and clarify the reason including analyzing muscle mass. Also other markers for nutritional status should be developed for patients with ADPKD, especially for female ADPKD patients.

In this study, we found that 23.5% of patients had nutritional problem (SGA score ≤6) even in early stage CKD (stage 1-3a). In addition, increased htTKLV was an independent risk factor after adjusting for kidney function by using a multivariate logistic regression model. Other nutritional biomarkers, such as prealbumin, insulin-like growth factor-1, or transferrin, were not assessed in this study. htTKLV could provide valuable information about nutritional status as well as the progression of disease, but it is cumbersome to measure with current methods. Therefore, developing new tools for the nutritional assessment of ADPKD patients is necessary, and such tools would be useful for improving long-term patient outcomes.

Even though this is the first observational study showing the impact of abdominal mass on nutritional status in ADPKD, it has several limitations. Relatively small numbers of patients in the low-SGA group may have undermined the statistical power, especially in females. Moreover, our hypothesis that the mass effect from enlarged liver and kidneys may be related to nutrition needs to be further verified by comparing with other non-ADPKD CKD groups, which is not possible for now because of lack of data on nutritional status in the early CKD stages. Also further comprehensive studies are needed to understand the factors affecting nutritional status in ADPKD patients including socioeconomic state and detailed food intake using diary. Although SGA is a well-validated method for nutritional assessment in patients with a range of conditions and is easy to perform, its ability to detect subtle changes in long-term nutritional status needs to be validated. Therefore, this study should be replicated in other larger cohorts, preferably in the form of a multicenter design including non-Asian populations. Also to improve the patients outcome eventually, more studies are needed to find out the possible effective therapies such as nutritional counseling and diet intervention to prevent and treat the malnutrition in ADPKD patients. This is a cross-sectional study but there are time interval between SGA measurement and CT scan as 12.5 ± 12.6 months. We used routinely perform CT scan to measure TKV, TLV and TKLV which are taken every other year in ADPKD outpatient clinic. Since the annual growth of PLD is about 0.9 to 3.2% we thought it was acceptable to use the information from the latest regular follow up CT image instead of taking new one at the time of nutritional assessment [28]. Because of this limitation, there were 4 patients who had renal aspiration and sclerotherapy between CT scan and SGA assessment. However when we analyzed data after excluding 4 patients, the similar result was seen and we reported whole patient’s data.

## Conclusions

In conclusion, detecting marginal malnutrition in patients in ADPKD outpatient clinics and initiating proper support can play an important therapeutic role, especially in patients who have decreased renal function or an increased htTKLV.

## Additional file

Additional file 1: Table S1. Baseline patient characteristics according to gender. TKLV, TKV, TLV, htTKLV, htTKV and htTLV are shown in median and interquartile range. BMI, body mass index; CKD, chronic kidney disease; eGFR, estimated glomerular filtration rate; htTKLV, height-adjusted total kidney and liver volume; htTKV, height-adjusted total kidney volume; htTLV, height-adjusted total liver volume; SGA; subjective global assessment, TKV, total kidney volume; TKLV; total kidney and liver volume; TLV, total liver volume. **Table S2.** Baseline patient characteristics according to nutritional status as evaluated by SGA (SGA 4 and 5 versus 6 and 7). TKLV, TKV, TLV, htTKLV, htTKV and htTLV are shown in median and interquartile range. BMI, body mass index; CKD, chronic kidney disease; eGFR, estimated glomerular filtration rates; htTKV, height-adjusted total kidney volume; htTKLV, height-adjusted total kidney and liver volume; htTLV, height-adjusted total liver volume; SGA; subjective global assessment, TKV, total kidney volume; TKLV; total kidney and liver volume; TLV, total liver volume. (DOCX 19.2 kb)

## Abbreviations

ADPKD: Autosomal dominant polycystic kidney disease
BMI: Body mass index
CKD: Chronic kidney disease
CT: Computed tomography
eGFR: Estimated glomerular filtration rate
ESRD: End-stage renal disease
htTKLV: Height-adjusted TKLV
htTKV: Height-adjusted TKV
htTLV: Height-adjusted TLV
IQR: Interquartile range
ROC: Receiver operating characteristic
sCr: Serum creatinine
SGA: Subjective global assessment
TKLV: Total kidney and liver volume
TKV: Total kidney volume
TLV: Total liver volume

## References

[1] Correia MI, Waitzberg DL (2003). The impact of malnutrition on morbidity, mortality, length of hospital stay and costs evaluated through a multivariate model analysis. Clin Nutr.

[2] Campbell KL, Ash S, Bauer J, Davies PSW (2007). Critical review of nutrition assessment tools to measure malnutrition in chronic kidney disease. Nutr Diet.

[3] Fouque D, Guebre-Egziabher F (2007). An update on nutrition in chronic kidney disease. Int Urol Nephrol.

[4] Lowrie EG, Huang WH, Lew NL (1995). Death risk predictors among peritoneal dialysis and hemodialysis patients: a preliminary comparison. Am J Kidney Dis.

[5] Leavey SF, Strawderman RL, Jones CA, Port FK, Held PJ (1998). Simple nutritional indicators as independent predictors of mortality in hemodialysis patients. Am J Kidney Dis.

[6] Djukanovic L, Lezaic V, Blagojevic R (2003). Co-morbidity and kidney graft failure-two main causes of malnutrition in kidney transplant patients. Nephrol Dial Transplant.

[7] Kopple JD (2001). National Kidney Foundation K/DOQI Clinical Practice Guidelines for Nutrition in Chronic Renal Failure. Am J Kidney Dis.

[8] Bae KT, Zhu F, Chapman AB (2006). Magnetic resonance imaging evaluation of hepatic cysts in early autosomal-dominant polycystic kidney disease: the Consortium for Radiologic Imaging Studies of Polycystic Kidney Disease cohort. Clin J Am Soc Nephrol.

[9] Kim H, Park HC, Ryu H (2015). Clinical Correlates of Mass Effect in Autosomal Dominant Polycystic Kidney Disease. PLoS One.

[10] Yang J, Ryu H, Han M, et al. Comparison of volume‐reductive therapies for massive polycystic liver disease in autosomal dominant polycystic kidney disease. Hepatol Res. 2016;46:183-91. 10.1111/hepr.12560 26190457

[11] Temmerman F, Missiaen L, Bammens B (2011). Systematic review: the pathophysiology and management of polycystic liver disease. Aliment Pharmacol Ther.

[12] Cnossen WR, Drenth JP (2014). Polycystic liver disease: an overview of pathogenesis, clinical manifestations and management. Orphanet J Rare Dis.

[13] Han N, Park HC, Kim H (2014). Hyperuricemia and deterioration of renal function in autosomal dominant polycystic kidney disease. BMC Nephrol.

[14] Adequacy of dialysis and nutrition in continuous peritoneal dialysis: association with clinical outcomes. Canada-USA (CANUSA) Peritoneal Dialysis Study Group. J Am Soc Nephrol 1996; **7**:198–207. 10.1681/ASN.V72198 8785388

[15] Kalantar-Zadeh K, Kopple JD, Block G, Humphreys MH (2001). A malnutrition-inflammation score is correlated with morbidity and mortality in maintenance hemodialysis patients. Am J Kidney Dis.

[16] Lawson JA, Lazarus R, Kelly JJ (2001). Prevalence and prognostic significance of malnutrition in chronic renal insufficiency. J Ren Nutr.

[17] Davies SJ, Phillips L, Griffiths AM, Naish PF, Russell GI (2000). Analysis of the effects of increasing delivered dialysis treatment to malnourished peritoneal dialysis patients. Kidney Int.

[18] Levey AS, Stevens LA (2010). Estimating GFR using the CKD epidemiology collaboration (CKD-EPI) creatinine equation: more accurate GFR estimates, lower CKD prevalence estimates, and better risk predictions. Am J Kidney Dis.

[19] O’Neill WC, Robbin ML, Bae KT (2005). Sonographic assessment of the severity and progression of autosomal dominant polycystic kidney disease: the Consortium of Renal Imaging Studies in Polycystic Kidney Disease (CRISP). Am J Kidney Dis.

[20] Cuppari L, Meireles MS, Ramos CI, Kamimura MA (2014). Subjective global assessment for the diagnosis of protein–energy wasting in nondialysis-dependent chronic kidney disease patients. J Ren Nutr.

[21] Figueiredo FA, Dickson ER, Pasha TM (2000). Utility of standard nutritional parameters in detecting body cell mass depletion in patients with end-stage liver disease. Liver Transpl.

[22] Chapman AB, Bost JE, Torres VE (2012). Kidney volume and functional outcomes in autosomal dominant polycystic kidney disease. Clin J Am Soc Nephrol.

[23] Miskulin DC, Abebe KZ, Chapman AB (2014). Health-related quality of life in patients with autosomal dominant polycystic kidney disease and CKD stages 1–4: a cross-sectional study. Am J Kidney Dis.

[24] Urata K, Hashikura Y, Ikegami T, Terada M, Kawasaki S (2000). Standard liver volume in adults. Transplant Proc.

[25] Campbell KL, Ash S, Bauer JD, Davies PS (2007). Evaluation of nutrition assessment tools compared with body cell mass for the assessment of malnutrition in chronic kidney disease. J Ren Nutr.

[26] Steiber A, Leon JB, Secker D (2007). Multicenter study of the validity and reliability of subjective global assessment in the hemodialysis population. J Ren Nutr.

[27] Fouque D, Pelletier S, Mafra D, Chauveau P (2011). Nutrition and chronic kidney disease. Kidney Int.

[28] Drenth JP, Chrispijn M, Nagorney DM, Kamath PS, Torres VE (2010). Medical and surgical treatment options for polycystic liver disease. Hepatology.

