# Supplementary material for: Total kidney and liver volume is a major risk factor for malnutrition in ambulatory patients with autosomal dominant polycystic kidney disease
Source: BMC Nephrol. 2017 Jan 14;18:22. doi: 10.1186/s12882-016-0434-0 (PMC5237538; doi:10.1186/s12882-016-0434-0)
Supplement: Additional file 1: Table S1. — Baseline patient characteristics according to gender. TKLV, TKV, TLV, htTKLV, htTKV and htTLV are shown in median and interquartile range. BMI, body mass index; CKD, chronic kidney disease; eGFR, estimated glomerular filtration rate; htTKLV, height-adjusted total kidney and liver volume; htTKV, height-adjusted total kidney volume; htTLV, height-adjusted total liver volume; SGA; subjective global assessment, TKV, total kidney volume; TKLV; total kidney and liver volume; TLV, total liver volume. Table S2. Baseline patient characteristics according to nutritional status as evaluated by SGA (SGA 4 and 5 versus 6 and 7). TKLV, TKV, TLV, htTKLV, htTKV and htTLV are shown in median and interquartile range. BMI, body mass index; CKD, chronic kidney disease; eGFR, estimated glomerular filtration rates; htTKV, height-adjusted total kidney volume; htTKLV, height-adjusted total kidney and liver volume; htTLV, height-adjusted total liver volume; SGA; subjective global assessment, TKV, total kidney volume; TKLV; total kidney and liver volume; TLV, total liver volume. (DOCX 19.2 kb) [file 12882_2016_434_MOESM1_ESM.docx]

**Table S1. Baseline patient characteristics according to gender.**

| Parameters | Male | Female | Total | P-value |
| --- | --- | --- | --- | --- |
| Number of patients | 150 | 138 | 288 |  |
| Age (years) | 47.4±13.6 | 49.4±10.4 | 48.3±12.2 | 0.155 |
| Height (cm) | 173.2±6.8 | 159.1±5.7 | 166.4±9.5 | *<0.001* |
| Weight (kg) | 72.1±10.9 | 58.1±7.7 | 65.4±11.8 | *<0.001* |
| BMI (kg/m^2^) | 23.9±2.9 | 22.9±2.7 | 23.4±2.8 | *0.004* |
| Hemoglobin (g/dL) | 14.3±1.6 | 12.7±1.0 | 13.5±1.5 | *<0.001* |
| eGFR (mL/min/1.73 m^2^) | 62.3±24.5 | 68.5±25.8 | 65.3±25.3 | *0.035* |
| Serum protein (g/dL) | 7.3±0.4 | 7.3±0.4 | 7.3±0.4 | 0.703 |
| Serum albumin (g/dL) | 4.4±0.3 | 4.3±0.4 | 4.4±0.3 | 0.421 |
| Total cholesterol (mg/dL) | 176.9±25.9 | 1,77.6±27.3 | 1,77.2±26.5 | 0.844 |
| Presence of hypertension | 134 (89.3%) | 104 (75.4%) | 238 (82.6%) | *0.002* |
| Presence of liver cysts | 101 (67.3%) | 126 (91.3%) | 227 (78.8%) | *<0.001* |
| TKLV (mL/m) | 3,133  [2,524-3,961] | 2,754  [2,061-4,021] | 2,981  [2,283-3,964] | 0.076 |
| TKV (mL/m) | 1,351  [880-2,080] | 1,004  [638-1,564] | 1,158  [685-1,881] | *0.003* |
| TLV (mL/m) | 1,691  [1,478-1,958] | 1,553  [1,288-2,356] | 1,647  [1,337-2,055] | 0.255 |
| htTKLV (mL/m) | 1,787  [1,428-2,340] | 1,730  [1,28- 2,511] | 1,776  [1,361-2,381] | 0.960 |
| htTKV (mL/m) | 796  [491-1,209] | 640  [405-992] | 697  [415-1,133] | 0.057 |
| htTLV (mL/m) | 978  [825-1,112] | 974  [807-1,471] | 977  [819-1,196] | 0.407 |
| SGA score | 6.7±0.6 | 6.6±0.6 | 6.6±0.6 | 0.197 |

TKLV, TKV, TLV, htTKLV, htTKV and htTLV are shown in median and interquartile range.

BMI, body mass index; CKD, chronic kidney disease; eGFR, estimated glomerular filtration rates; htTKV, height-adjusted total kidney volume; htTKLV, height-adjusted total kidney and liver volume; htTLV, height-adjusted total liver volume; SGA, subjective global assessment; TKV, total kidney volume; TKLV; total kidney and liver volume; TLV, total liver volume.

**Table S2. Baseline patient characteristics according to nutritional status as evaluated by SGA (SGA 4 and 5 versus 6 and 7)**

| Parameters | Mildly to moderately malnourished  (SGA 4 and 5) | At risk to well nourished  (SGA 6 and 7) | P-value |
| --- | --- | --- | --- |
| Number of patients | 21 (7.3%) | 267 (92.7%) |  |
| Female | 9 (42.9%) | 129 (48.3%) | 0.658 |
| Age (years) | 53.4±11.1 | 47.9±12.2 | *0.046* |
| Height (cm) | 164.0±7.9 | 166.6±9.6 | 0.285 |
| Weight (kg) | 59.1±8.7 | 65.9±11.9 | *0.021* |
| BMI (kg/m^2^) | 22.0±2.7 | 23.6±2.8 | *0.013* |
| Hemoglobin (g/dL) | 12.8±1.1 | 13.6±1.5 | *0.003* |
| eGFR (mL/min/1.73 m^2^) | 51.3±23.2 | 66.3±23.2 | *0.009* |
| Protein (g/dL) | 7.4±0.4 | 7.3±0.4 | 0.476 |
| Albumin (g/dL) | 4.4±0.3 | 4.4±0.3 | 0.911 |
| Total cholesterol (mg/dL) | 180.3±30.2 | 177.0 ±26.3 | 0.585 |
| Presence of hypertension | 21 (100%) | 217 (81.3%) | *0.016* |
| Presence of liver cysts | 17 (81%) | 210 (78.7%) | 0.530 |
| TKLV (mL) | 4,581 [2,698-7,899] | 2,918 [2,273-3,851] | *0.003* |
| TKV (mL) | 2,090 [817-2,801] | 1,134 [674-1,750] | *0.018* |
| TLV (mL) | 2,039 [1,336-4,728] | 1,639 [1,338-1,969] | *0.049* |
| htTKLV (mL/m) | 2,622 [1,719-4,906] | 1747 [1354, 2308] | *0.002* |
| htTKV (mL/m) | 1282 [524, 1574] | 686 [406, 1079] | *0.013* |
| htTLV (mL/m) | 1299 [830, 2831] | 964 [819, 1171] | *0.036* |

TKLV, TKV, TLV, htTKLV, htTKV and htTLV are shown in median and interquartile range.

BMI, body mass index; CKD, chronic kidney disease; eGFR, estimated glomerular filtration rates; htTKV, height-adjusted total kidney volume; htTKLV, height-adjusted total kidney and liver volume; htTLV, height-adjusted total liver volume; SGA; subjective global assessment, TKV, total kidney volume; TKLV; total kidney and liver volume; TLV, total liver volume.
